# Supplementary material for: A prospective cohort study of cochlear implantation as a treatment for tinnitus in post-lingually deafened individuals
Source: Commun Med (Lond). 2024 Dec 19;4:274. doi: 10.1038/s43856-024-00692-8 (PMC11659473; doi:10.1038/s43856-024-00692-8)
Supplement: Supplementary file 2 — Supplementary Materials [file 43856_2024_692_MOESM2_ESM.pdf]

Supplementary Materials for

A prospective cohort study of cochlear implantation as treatment for tinnitus and related symptoms in post-lingually deafened individuals

Qian Wang, Michelle R. Kapolowicz, Jia-Nan Li, Shuo Han, Fei Ji, Wei-Dong Shen, Fang-Yuan Wang, Wei Chen, Wei-Wei Guo, Chi Zhang, Ri-Yuan Liu, Miao Zhang, Meng-Di Hong, Ai-Ting Chen, Fan-Gang Zeng, Shi-Ming Yang

The PDF file includes:

Figure S1. Audiograms for the Tinnitus and No-Tinnitus cohort.

Table S1. Statistical analysis of audiograms.

Table S2. Statistical analysis of tinnitus loudness.

Table S3. Statistical analysis of Tinnitus Handicap Inventory (TFI).

Table S4. Effects of one or two implants on unilateral or bilateral tinnitus.

Figure S1. Audiograms for the Tinnitus and No-Tinnitus cohort. Pre-surgical hearing thresholds (dB HL) as a function of audio frequency from 0.125 to 8 kHz. Left panel=Left ear (crosses) and Right panel=Right ear (circles); Tinnitus group=red and No-Tinnitus group=blue. Error bars represent 95 confidence intervals. Mixed ANOVA (Table S1) showed no difference in the left or right ear between the two groups.

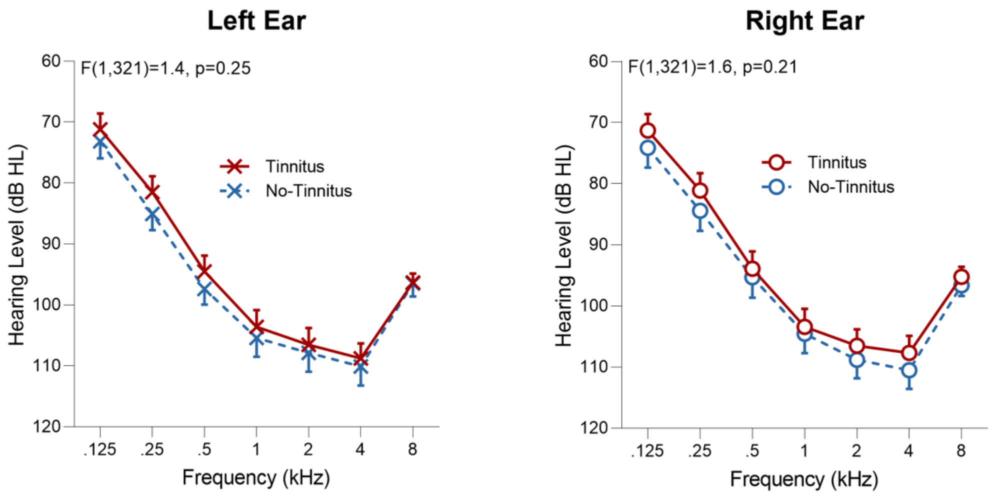

Table S1. Statistical analysis of audiograms. Two-way mixed ANOVA shows the main effects of frequency, group and their interactions in the left (top section) and right (bottom) ears.

**Table S1. Audiograms for the Tinnitus and No-Tinnitus groups for each ear**							
**Left ear: (Two-way mixed effects ANOVA)**							
Main effects	F	df	p				
Frequency	434.9	3, 8	**$1x10^{-15}$**				
Group	1.4	1, 321	0.2461				
Frequency x group	0.7	6, 1926	0.6279				
Sidak's multiple comparisons test (Tinnitus vs No-Tinnitus)							
Frequency (kHz)	.125	.25	.5	1	2	4	8
t	1.0	1.9	1.5	0.9	0.6	0.7	0.1
df	271	283	291	261	261	235	212
Adjusted p	0.9254	0.3447	0.6012	0.9668	0.9952	0.9934	0.9999
Mean difference	-2.0	-3.6	-2.9	-1.8	-1.3	-1.3	-0.2
Lower 95% CI of mean difference	-7.2	-8.7	-7.9	-7.5	-7.0	-6.8	-3.7
Upper 95% CI of mean difference	3.3	1.5	2.1	3.8	4.4	4.1	3.4
**Right ear: (Two-way mixed effects ANOVA)**							
Main effects	F	df	p				
Frequency	377.8	2, 755	**$1x10^{-15}$**				
Group	1.6	1, 321	0.2068				
Frequency x group	0.4	6, 1926	0.8841				
Sidak's multiple comparisons test (Tinnitus vs No-Tinnitus)							
Frequency (kHz)	.125	.25	.5	1	2	4	8
t	1.3	1.5	0.6	0.5	1.1	1.3	1.2
df	247	257	253	272	261	267	270
Adjusted p	0.7497	0.6197	0.9954	0.9984	0.8809	0.7556	0.8655
Mean difference	-2.9	-3.3	-1.4	-1.1	-2.3	-2.8	-1.4
Lower 95% CI of mean difference	-8.6	-9.2	-7.4	-7.1	-7.8	-8.5	-4.7
Upper 95% CI of mean difference	2.9	2.6	4.7	4.8	3.2	2.9	1.9

Table S2. Statistical analysis of tinnitus loudness. The top section shows the Tinnitus cohort with reduced loudness at post-implant activation. The bottom section shows only descriptive statistics for the No-Tinnitus cohort because most values are “0”; the mean difference was 0.1 over the seven post-activation times for the No-Tinnitus cohort.

**Table S2. Time course for tinnitus loudness after CI activation**							
**Tinnitus: Implant on (One-way repeated measures ANOVA)**							
Main effect	F	df	p				
Time	328.6	3, 992	**$1x10^{-15}$**				
Dunnett’s multiple comparisons test (baseline vs each time point)							
Time (months)	0	1	2	3	6	12	24
q	19.9	21.8	23.0	23.5	25.1	25.0	24.8
df	349	349	349	349	349	349	349
Adjusted p	**$1x10^{-13}$**	**$1x10^{-13}$**	**$1x10^{-13}$**	**$1x10^{-13}$**	**$1x10^{-13}$**	**$1x10^{-13}$**	**$1x10^{-13}$**
Mean difference	-1.6	-1.8	-2.0	-2.2	-2.4	-2.4	-2.4
Lower 95% CI of mean difference	-1.8	-2.0	-2.2	-2.3	-2.6	-2.7	-2.7
Upper 95% CI of mean difference	-1.4	-1.6	-1.8	-2.0	-2.1	-2.2	-2.2
**No-Tinnitus: (Descriptive statistics for difference from baseline scores reported since most values are “0”)**							
Time (months)	0	1	2	3	6	12	24
Mean	0.2	0.1	0.1	0.1	0.1	0.1	0.1
Lower 95% CI of mean	0.1	0.1	0.0	0.0	0.0	0.0	0.0
Upper 95% CI of mean	0.2	0.2	0.2	0.2	0.2	0.2	0.2

Table S3. Statistical analysis of Tinnitus Handicap Inventory (TFI). The top section shows the Tinnitus cohort with reduced THI at post-implant activation. The bottom section shows only descriptive statistics for the No-Tinnitus cohort because most values are "0"; the mean difference was 1.7 over the seven post-activation times for the No-Tinnitus cohort.

**Table S3. Time course for tinnitus handicap inventory (THI) after CI activation**							
**Tinnitus: Implant on (One-way repeated measures ANOVA)**							
Main effect	F	df	p				
Time	239.2	2, 319	**$1x10^{-15}$**				
Dunnett's multiple comparisons test (baseline vs each time point)							
Time (months)	0	1	2	3	6	12	24
q	2.1	9.3	11.5	16.4	17.0	16.8	16.9
df	211	211	211	211	211	211	211
Adjusted p	0.1590	**$1x10^{-15}$**	**$1x10^{-15}$**	**$1x10^{-15}$**	**$1x10^{-15}$**	**$1x10^{-15}$**	**$1x10^{-15}$**
Mean difference	-1.0	-5.4	-7.4	-14.7	-19.6	-21.0	-21.2
Lower 95% CI of mean difference	-2.3	-7.0	-9.0	-17.1	-22.6	-24.3	-24.5
Upper 95% CI of mean difference	0.2	-3.9	-5.7	-12.3	-16.5	-17.7	-17.9
**No-Tinnitus: (Descriptive statistics for difference from baseline scores reported since most values are "0")**							
Time (months)	0	1	2	3	6	12	24
Mean difference	2.8	2.0	1.5	1.5	1.4	1.3	1.4
Lower 95% CI of mean difference	1.9	0.9	0.2	0.1	0.0	0.0	0.0
Upper 95% CI of mean difference	3.6	3.0	2.7	2.9	2.7	2.7	2.9

Table S4. Effects of one or two implants on unilateral or bilateral tinnitus. Table S4 shows the statistical analyses from the data presented in Fig 3A, 3B, and 3C, respectively, regarding the effects of one or two implants on unilateral or bilateral tinnitus.

**Table S4. Effects of one or two implants on unilateral or bilateral tinnitus**							
**Fig. 3A. Effect of one implant on bilateral tinnitus loudness: Same vs opposite side (Two-way mixed effects ANOVA)**							
Main effects	F	df	p				
Time	60.3	2, 562	**p=1x10^{-15}**				
Implanted side	84.3	1, 240	**p=1x10^{-15}**				
Time x implanted side	1.7	6, 1440	0.1154				
Sidak's multiple comparisons test							
Time (months)	0	1	2	3	6	12	24
t	9.5	9.1	9.5	9.2	7.3	7.5	7.4
df	214	223	227	228	232	232	233
Adjusted p	**p=1x10^{-15}**	**p=1x10^{-15}**	**p=1x10^{-15}**	**p=1x10^{-15}**	**p=3x10^{-11}**	**p=1x10^{-11}**	**p=2x10^{-11}**
Mean difference	-1.6	-1.6	-1.8	-1.9	-1.6	-1.7	-1.7
Lower 95% CI of mean difference	-2.0	-2.0	-2.3	-2.4	-2.2	-2.3	-2.3
Upper 95% CI of mean difference	-1.1	-1.1	-1.3	-1.3	-1.0	-1.1	-1.1
**Fig. 3B. Effect of one implant on unilateral tinnitus loudness: Same vs opposite side (Two-way mixed effects ANOVA)**							
Main effects	F	df	p				
Time	23.5	3, 178	**p=3x10^{-12}**				
Implanted side	6.8	1, 64	**0.0112**				
Time x implanted side	4.2	6, 384	**0.0004**				
Sidak's multiple comparison test							
Time (months)	0	1	2	3	6	12	24
t	4.1	3.0	2.8	1.7	1.9	2.2	1.8
df	64	64	64	63	62	63	63
Adjusted p	**0.0008**	**0.0279**	0.0521	0.5089	0.3516	0.2119	0.4552
Mean difference	-1.5	-1.2	-1.0	-0.7	-0.8	-0.8	-0.7
Lower 95% CI of mean difference	-2.5	-2.2	-2.0	-1.8	-1.9	-1.8	-1.9
Upper 95% CI of mean difference	-0.5	-0.1	0.0	0.5	0.3	0.2	0.4
**Fig. 3C. Effects of bilateral and unilateral implant on tinnitus (Two-way mixed effects ANOVA)**							
Main effects	F	df	p				
Time	26.7	3, 565	**p=1x10^{-15}**				
Bilateral/unilateral	0.2	1, 195	0.6722				
Time x bilateral/unilateral	1.2	6, 1170	0.2800				
Sidak's multiple comparison test							
Time (months)	0	1	2	3	6	12	24
t	0.1	0.1	0.0	1.1	0.6	1.1	0.5
df	131	90	79	87	94	85	97
Adjusted p	0.9999	0.9999	0.9999	0.8778	0.9976	0.9028	0.9994
Mean difference	0.0	0.0	0.0	0.3	0.1	0.3	0.1
Lower 95% CI of mean difference	-0.5	-0.6	-0.6	-0.4	-0.5	-0.4	-0.5
Upper 95% CI of mean difference	0.4	0.6	0.6	0.9	0.8	1.0	0.8
