# Supplementary material for: A prospective cohort study of cochlear implantation as a treatment for tinnitus in post-lingually deafened individuals
Source: Commun Med (Lond). 2024 Dec 19;4:274. doi: 10.1038/s43856-024-00692-8 (PMC11659473; doi:10.1038/s43856-024-00692-8)
Supplement: Supplementary file 3 — Description of Additional Supplementary Files [file 43856_2024_692_MOESM3_ESM.pdf]

Description of Additional Supplementary Files

File name: Supplementary Data 1

File description: Numerical values for Figures 2, 3 and 4
